# Myoelectric Pattern Recognition Performance Enhancement Using Nonlinear Features

**DOI:** 10.1155/2022/6414664

**Published:** 2022-04-29

**Authors:** Md. Johirul Islam, Shamim Ahmad, Fahmida Haque, Mamun Bin Ibne Reaz, Mohammad A. S. Bhuiyan, Khairun Nisa' Minhad, Md. Rezaul Islam

**Affiliations:** ^1^Department of Electrical and Electronic Engineering, University of Rajshahi, Rajshahi 6205, Bangladesh; ^2^Department of Physics, Rajshahi University of Engineering and Technology, Rajshahi 6204, Bangladesh; ^3^Department of Computer Science and Engineering, University of Rajshahi, Rajshahi 6205, Bangladesh; ^4^Department of Electrical, Electronic and Systems Engineering, Universiti Kebangsaan Malaysia, Bangi 43600, Selangor, Malaysia; ^5^Department of Electrical and Electronic Engineering, Xiamen University Malaysia, Bandar Sunsuria, Sepang 43900, Selangor, Malaysia

## Abstract

The multichannel electrode array used for electromyogram (EMG) pattern recognition provides good performance, but it has a high cost, is computationally expensive, and is inconvenient to wear. Therefore, researchers try to use as few channels as possible while maintaining improved pattern recognition performance. However, minimizing the number of channels affects the performance due to the least separable margin among the movements possessing weak signal strengths. To meet these challenges, two time-domain features based on nonlinear scaling, the log of the mean absolute value (LMAV) and the nonlinear scaled value (NSV), are proposed. In this study, we validate the proposed features on two datasets, the existing four feature extraction methods, variable window size, and various signal-to-noise ratios (SNR). In addition, we also propose a feature extraction method where the LMAV and NSV are grouped with the existing 11 time-domain features. The proposed feature extraction method enhances accuracy, sensitivity, specificity, precision, and F1 score by 1.00%, 5.01%, 0.55%, 4.71%, and 5.06% for dataset 1, and 1.18%, 5.90%, 0.66%, 5.63%, and 6.04% for dataset 2, respectively. Therefore, the experimental results strongly suggest the proposed feature extraction method, for taking a step forward with regard to improved myoelectric pattern recognition performance.

## 1. Introduction

**Figure 1:**
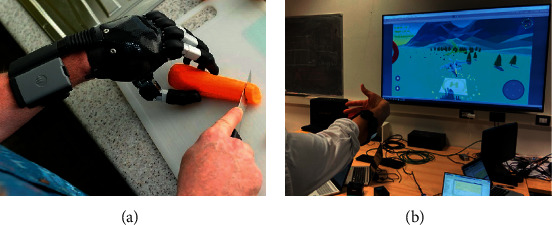
Applications of myoelectric pattern recognition. (a) Prosthetic hand source: https://mcopro.com/blog/resources/arm-hand-prosthetics/ (accessed on 05 Apr. 2022). (b) Game controller (source: https://www.miro.ing.unitn.it/emg-remote-control-of-a/ (accessed on 05 Apr. 2022).

From the perspective of performing regular activities after limb loss or from the view of people born with congenital defects, artificial limbs or prostheses are very helpful [1]. Many modern prostheses, such as i-Limb [2], Cyberhand [3], and Yokoi Hand [4], use EMG signals to control multiple degrees of freedom of prosthesis movements since the EMG signal reflects the activity of a muscle corresponding to a movement [5, 6]. Electromyography is a technique that senses the bioelectrical potential, also known as the EMG signal, from a target muscle or group of muscles with the help of a surface electrode or needle electrode when these muscles are neurologically activated [7–10]. Generally, myoelectric pattern recognition employed in prosthetic hand and game controller (Figure 1) uses surface EMG because it is noninvasive and convenient for long-term data acquisition [11–13].

In addition to EMG signal, force myography (FMG), a noninvasive technique for measuring the pressure patterns between the underlying muscle and the pressure sensor during muscle contraction, is also used for upper limb prosthetic control [14–17]. Also, mechanomyography (MMG) is another alternative technique that measures vibrational characteristics during muscle contraction employing an accelerometer or microphone [18–20]. But the frequency spectrum of EMG is wide compared to FMG and MMG and carries more information corresponding to a muscle contraction [21]. In myoelectric pattern recognition, the features, the vital components of myoelectric pattern recognition, are extracted from the EMG signal. An efficient feature extraction technique derives unique information about each movement hidden in the raw EMG signal [22, 23]. To improve the EMG pattern recognition performance and ensure more degree of freedom, large numbers of time-domain, frequency-domain, and time-frequency-domain EMG features have been reported [24, 25]. Popular time-domain features are found in many studies; these include the mean absolute value (MAV), waveform length (WL), number of zero crossings (ZC), and slope sign changes (SSC), which are mentioned in [26]; the variance (VAR), complexity (COM), and mobility (MOB), which are reported in [27]; and the Wilson amplitude (WAMP), log detector (LOG), and autoregressive coefficients (ARs), which are described in [28]. In addition, some other time-domain features are the myopulse percentage rate (MYOP) [29], skewness (SKW) [30], difference absolute mean value (DAMV) [31], difference absolute standard deviation value (DASDV) [31], root mean square (RMS) [24], and maximum fractal length (MFL) [32]. Thereafter, the most commonly used frequency-domain features are the mean frequency (MNF) and mean power (MNP) proposed in [33, 34] and the median frequency (MDF), frequency ratio (FR), spectral moment (SM), total power (TTP), and variance of the central frequency (VCF) given in [35]. Moreover, short-time Fourier transforms or wavelet transforms extract features both in time and frequency domains. However, in the literature, time-domain features are used more frequently than frequency- or time-frequency-domain features since these features do not require any transformations and hence large computing resources, such as processing power and memory [25, 36]. Consequently, this research is carried out to determine a further contribution to the time-domain feature set.

Khushaba et al. [37] proposed six time-domain features and spectrum correlations between each pair of channels for arm position-invariant EMG pattern recognition using a seven-channel EMG signal. Furthermore, Al-Timemy et al. [38] extended the work of Khushaba et al. [37] and introduced six modified time-domain features to improve EMG pattern recognition performance against muscle force variations, where eight channels were employed to collect EMG signals. Thereafter, Khushaba et al. [39] modified and upgraded their pilot work and proposed seven time-domain features that were validated over five EMG datasets. In these EMG datasets, the number of channels varied from eight to one hundred twenty-eight. Recently, Asogbon et al. [40] described five time-domain features to resolve the effects of both limb position and muscle force variation simultaneously, where they included eight EMG signal channels. The major limitation of a large number of existing features is that these features are unable to address all the requirements for a given application since a specific feature is effective for a specific type of application with a specific arrangement [26, 28, 29]. Again, all the authors mentioned above used multichannel EMG signals with a minimum of seven channels to validate their proposed features. However, multichannel myoelectric pattern recognition increases the computational cost and device cost [41]. In addition, some of the proposed features are applicable only for multichannel EMG systems [42–44]. Thus, the existing features validated for multichannel systems may not be effective for few numbers of channels. Again, a myoelectric pattern recognition system using the fewest possible channels lacks spatial information, which in turn decreases the separation margins among the movements [45–47]. Usually, this problem occurs among movements possessing relatively weaker signals than the others due to the overlapping of activated muscles, narrow muscles, muscle activations with low contraction forces, etc. [32, 48].

In this context, to minimize these limitations, we have proposed two time-domain features, namely, the LMAV and NSV, which should improve the separation margins among the movements when the number of channels used was two. These proposed features are based on the nonlinear scaling, log, and cubic root of the signal amplitude. The LMAV produced relatively higher discrimination among weak signals than among strong signals. In addition, the NSV measured the nonlinear deviation of each sample value from its linear mean absolute value, focusing on the instantaneous amplitude of a weak signal. These proposed features maximized the margins among the least separable movements. Consequently, these proposed features improved the EMG pattern recognition performance of a model in terms of accuracy, sensitivity, specificity, precision, and F1 score when these two features were grouped with the existing four feature extraction methods considered in this study. This performance improvement means that the proposed LMAV and NSV add some new information to the existing feature extraction methods which in turn contribute to improving EMG pattern recognition performance. Moreover, the LMAV and NSV showed strength over variable window size, variable SNR, movement-wise performance enhancement and datasets. In addition, a combined feature extraction method was also proposed in this study, where the LMAV and NSV were grouped with existing 11 time-domain features, including the WL, WAMP, SSC, ZC, MOB, COM, SKW, and four autoregressive coefficients. The proposed feature extraction method achieved the highest EMG pattern recognition performance in terms of all performance evaluating parameters.

In terms of EMG pattern recognition, many classifiers have been used in recent studies. These are convolutional neural networks (CNNs) [49, 50], linear discriminant analysis (LDA) [51], artificial neural networks (ANNs) [52], fuzzy methods [53], support vector machines (SVMs) [54, 55], and *k*-nearest neighbours (KNNs) [56]. Among these methods, the CNN provides very strong EMG recognition performance but is impossible to implement in cheap hardware for real-time operation [57]. Therefore, to minimize the hardware cost and obtain an acceptable level of performance, we used LDA, an SVM, and the KNN algorithm as classifiers, all of which are widely used for these types of applications [39, 51, 58]. Furthermore, the resulting EMG pattern recognition performance was investigated over two datasets (newly collected and standard datasets with the same arrangements) to validate our results.

The rest of the sections are structured as follows.  Section 2 describes the EMG datasets, proposed features, scatter plots, and EMG pattern recognition method.  Section 3 presents the experimental results, where the resulting performances are evaluated and compared with those of other considered feature extraction methods.  Section 4 investigates the reasons for the obtained performance enhancement, and Section 5 concludes with the overall experimental results.

## 2. Methodology

### 2.1. EMG Data Collection

In this study, we employed two EMG datasets, where datasets 1 and 2 were collected using our EMG signal acquisition system and a public dataset from online, respectively.

#### 2.1.1. Acquisition of Dataset 1

**Figure 2:**
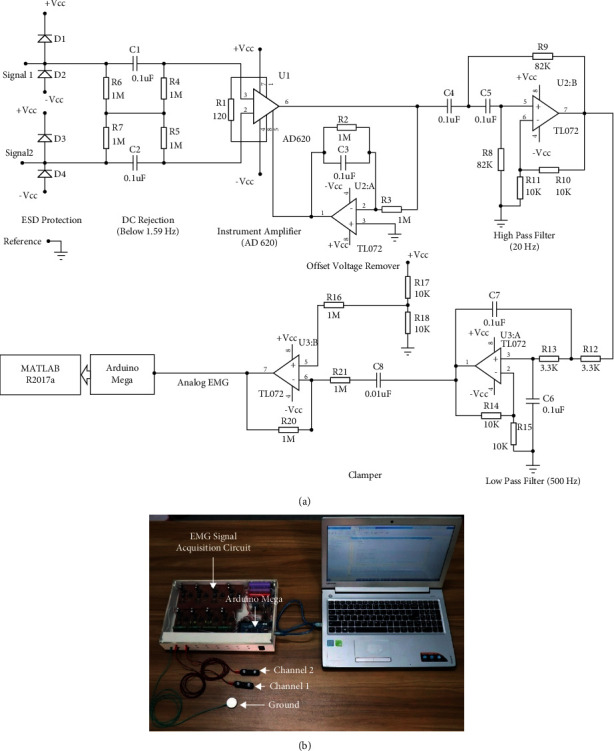
The EMG data acquisition system. (a, b) The schematic circuit diagram and an EMG signal acquisition system.

For the acquisition of this EMG dataset, we employed an EMG signal acquisition system.  Figure 2(a) shows the schematic circuit diagram of a single-channel bipolar EMG signal acquisition system. It consists of several functional blocks, including electrostatic discharge (ESD) protection, DC rejection, an instrument amplifier, a high-pass and low-pass filter, and a clamper. In this system, the ESD unit provided a low-resistance path for a high electrostatic charge on the human body and protects sophisticated devices [59]. In addition to electrostatic charge, the raw EMG signal also possesses DC half-cell potential produced on the electrode-skin interface and its amplitude is enough to saturate a high gain instrument amplifier.

So, the DC offset voltage was removed by passing the signal through a DC rejection circuit (also known as a balanced AC coupling network). It is mainly a differential high-pass filter whose cutoff frequency lies near to DC frequency. The advantage of this filter is that it offers a bias path without any ground connection resulting in a high common-mode rejection ratio (CMRR) of the instrument amplifier [60]. Thereafter, we used an instrument amplifier integrated circuit (AD620) for the differential amplification of the raw EMG signal [61]. In addition, there exists an offset voltage with regard to the instrument amplifier itself. To eliminate the offset voltage of the instrument amplifier, we employed a unity-gain inverting amplifier, and the output of this amplifier was used to ground the instrument amplifier [62]. During muscle contraction, the electrode shifts slightly which generate a noise also known as movement artefacts whose cutoff frequency lies between 0 Hz and 20 Hz [63]. So, a second-order high-pass filter of 20 Hz was employed to remove it. Finally, we employed a second-order low-pass filter of 500 Hz to eliminate high-frequency noise [63]. Then, we used a positive clamper circuit to shift the DC level to 2.5 V. Finally, we employed an Arduino Mega for digitalizing the EMG signal at a resolution of 10 bits with a sampling frequency of 2000 Hz.

**Figure 3:**
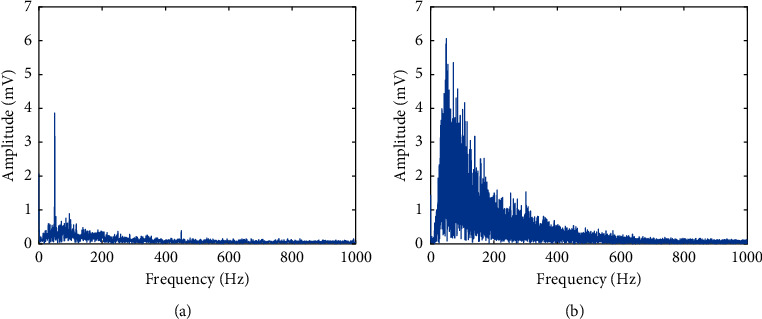
The frequency spectrum of EMG signal acquisition system: (a) noise and (b) EMG signal.

**Figure 4:**
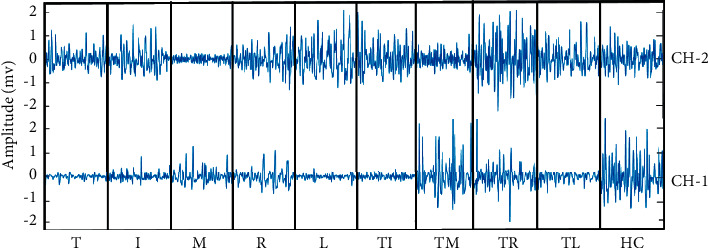
The EMG signal of dataset 2 in the time domain for ten-finger movements collected from two channels.

Figure 2(b) shows the implementation of the multichannel bipolar EMG signal acquisition system, where the circuit shown in Figure 2(a) was repeated for all channels. In this system, we used an MFI bar electrode made in the USA as a surface electrode. In this device, the average EMG signal strength (RMS) during no-movement condition was considered as noise and the value found was 11.9 mV where the system gain was 1652 (gain of instrument amplifier × gain of high pass filter × gain of low pass filter = 413 × 2 × 2).  Figure 3(a) shows the frequency spectrum of noise where the dominant noise comes from power line artefacts and its harmonics; however, these can be minimized by employing a digital notch filter [63]. In addition to the power line artefact, Figure 3(a) also indicates that the noise includes additive white Gaussian noise (AWGN) and some low amplitude EMG signal. Again, Figure 3(b) shows the frequency spectrum of a movement (mixed with noise) which was very high in amplitude relative to the noise, but it varied with the movements (Figure 4) and muscle force levels. In this dataset, the average SNR for medium force level was calculated using (1). First, noise (RMS) was eliminated from the RMS value of the raw EMG signal and the average SNR value for each subject was calculated in dB. In this study, the obtained SNR values varied across all subjects and lied between 5 dB and 23 dB.

(1) $$\begin{array}{c} SNR=20\;\log_{10}\left(\frac{\sqrt{\mathrm{Raw}\;\mathrm{EMG}\;{\text{Signal}}_{RMS}^{2}-{\text{Noise}}_{RMS}^{2}}}{{\text{Noise}}_{RMS}}\right). \end{array}$$

**Figure 5:**
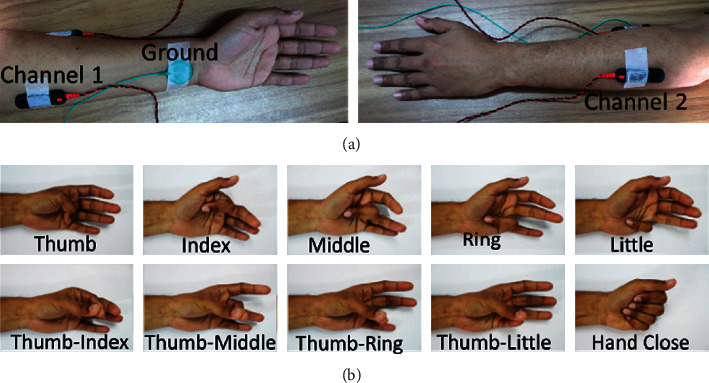
The EMG signal acquisition settings. (a, b) The electrode placement and the finger movements.

For this dataset, we collected a two-channel EMG signal: channel 1 collects the EMG signal from the flexor digitorum superficialis, flexor digitorum profundus, and remote flexor pollicis longus, and channel 2 collects the EMG signal from the extensor digitorum communis, extensor digiti minimi, and remote extensor pollicis longus. In addition, the ground electrode was placed on the wrist, as shown in Figure 5. To ensure proper contact between the electrodes and skin, the electrodes were attached to the skin through an adhesive conductive gel. However, ten intact-limbed subjects aged between 25 and 55 years were engaged to perform five individual finger movements, thumb (T), index (I), middle (M), ring (R), little (L), and five combined finger movements, i.e., thumb-index (TI), thumb-middle (TM), thumb-ring (TR), thumb-little (TL), and hand closing (HC) movements, as shown in Figure 5(b). During this signal acquisition phase, we informed all the participants about the objective of the research, and they provided us with their written consent in this regard. Ethical approval was provided by the Faculty of Engineering, University of Rajshahi, Bangladesh, to perform this study. During data collection, the subjects sat on a handled chair to place their hands freely. During the recording process, each of the finger movements was repeated six times with a duration of five seconds. In addition, the subjects were relaxed for 5 to 10 seconds between successive movements.

#### 2.1.2. Description of Dataset 2

**Figure 6:**
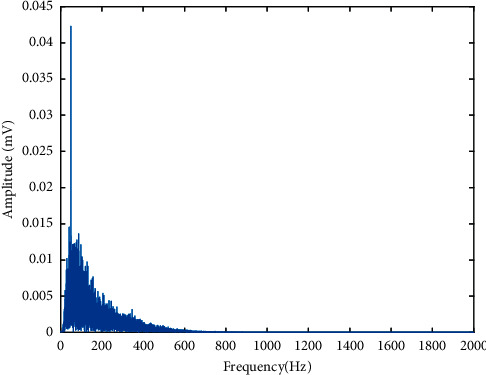
The frequency spectrum of an EMG signal of dataset 2.

In this study, we also collected the same dataset as dataset 1 to validate the result obtained from the Khushaba website [64]; here, Delsys DE 2.x series EMG sensors from Bagnoli Desktop EMG Systems were used for data acquisition. In this dataset, there were eight subjects, including six males and two females aged 20 to 35 years. Each subject performed five individual movements (T, I, M, R, and L) and five combined finger movements (TI, TM, TR, TL, and HC) as shown in Figure 5(b) providing six trials for each movement where each trial was five seconds long in duration. The EMG signal was sampled at 4000 Hz and digitalized with a 12 bit resolution using a National Instruments BNC-2090. In this dataset, the signal was in raw condition with the significant frequency spectrum of 20 Hz to 500 Hz (Figure 6).

### 2.2. Feature Extraction

An EMG signal is composed of hidden unique information for each movement. Feature extraction methods are employed to obtain as few features as possible, to obtain the most effective feature(s) or to derive a new feature for a particular application. The performance of EMG pattern recognition strongly depends on proper feature selection rather than the classification algorithms used [29, 35].

#### 2.2.1. The Proposed Time-Domain Features

In our studies, we have proposed two time-domain features as described below.

The first proposal was the log of the mean absolute value (LMAV), which is mainly a nonlinear scaling of the mean absolute value. We found that the LMAV for a given window highly discriminates or focuses on a low amplitude EMG signal; for this reason, it was expected that it could provide better performances than currently used features. The LMAV is expressed mathematically as

(2) $$\begin{array}{c} \mathrm{LMAV=}{\text{log}}_{e}\left(\frac{1}{N}\sum_{\mathrm{i=1}}^{N}\left|x_{i}\right|\right), \end{array}$$

where *N* represents the size of the window and *x*_*i*_ denotes the *i*th sample within the corresponding window.

**Figure 7:**
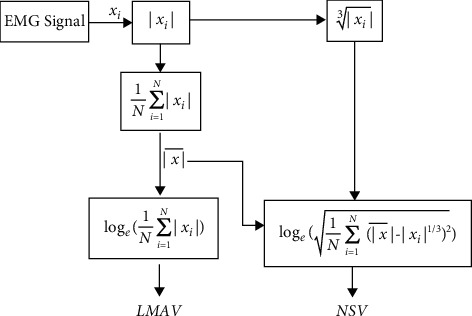
The feature extraction procedure of the proposed time-domain features.

The second proposal was the nonlinear scaled value (NSV). This NSV is based on nonlinear scaling operations. The NSV measures the nonlinear deviation of each sample value from its linear mean absolute value to focus on the instantaneous amplitude of a weak EMG signal. It also emphasizes discriminating low amplitude EMG signals rather than high amplitude signals.

(3) $$\begin{array}{c} NSV={\text{log}}_{e}\left(\sqrt{\frac{1}{N}\sum_{i=1}^{N}\left(\bar{\left|x\right|}-{\left|x_{i}\right|}^{1/3}\right){}^{2}}\right), \end{array}$$

where $\bar{\left|x\right|}\;$ represents the mean absolute value for a window of size *N*. However, the feature extraction steps are summarized in Figure 7.

#### 2.2.2. The Feature Extraction Methods

Time-domain features are widely used because they do not require any mathematical transformations or modifications; as a result, they ensure low time consumption in pattern recognition tasks [29]. In this study, we used five popular time-domain feature extraction methods, where each method includes several features. These include the following.

Huang et al. [65] used seven features (FS1) and six autoregressive coefficients along with the RMS value.

Du et al. [66] used six time-domain features (FS2) that include the integration of the EMG (IEMG), WL, WAMP, ZC, SSC, and VAR.

Time-dependent power spectrum descriptors (FS3) [38] introduce six time-domain features: the root squared zero-order, second-order, and fourth-order moments; an irregularity factor; sparseness; and the waveform length ratio. In this study, we used six features directly.

The temporal-spatial descriptors (FS4) [39] describe seven time-domain features: the root squared zero-order, second-order, and fourth-order moments; an irregularity factor; sparseness; the coefficient of variation; and the Teager-Kaiser energy operator. In this research, we employed seven features only.

**Figure 8:**
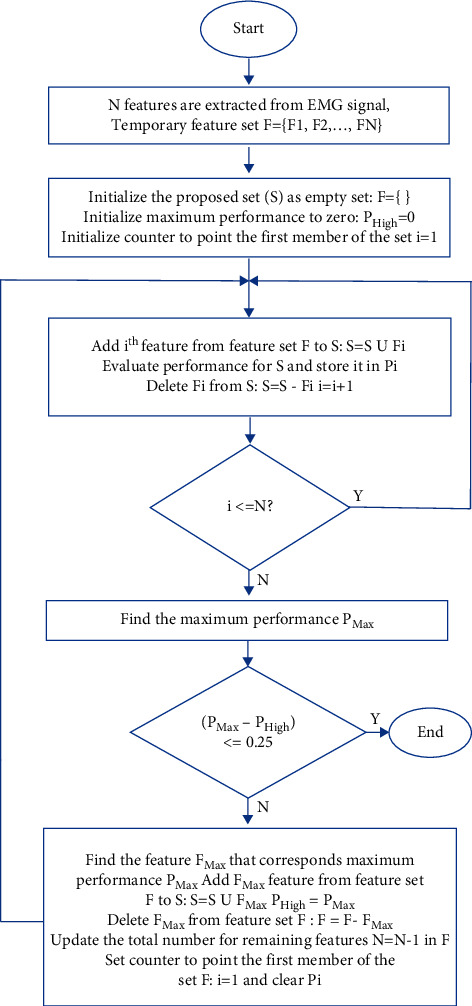
The forward feature selection algorithm.

In this study, we have proposed a combined feature extraction method which was selected from the proposed LMAV and NSV, and the existing time-domain features [26–32]. For the selection of these features, we used the forward feature selection algorithm [67] shown in Figure 8, which was evaluated across 32 time-domain features. In this feature selection, first, we selected the highest performing feature among all the features. Then, we grouped the best performing feature with each of the remaining features one by one and find the group that yields the strongest performance. However, we considered a new feature from the highest performing group only when it satisfied the condition of a minimum performance enhancement of 0.25. Thus, the algorithm selected 13 features which were denoted as the proposed feature extraction method, including the proposed LMAV and NSV along with existing 11 time-domain features, i.e., WL, WAMP, SSC, ZC, MOB, COM, and SKW, and four autoregressive coefficients. Here, the WAMP gives the signal energy, the WL provides the collective length of the EMG waveform, the SSC and ZC describe indirect frequency information, the MOB represents the mean frequency or the proportion of the standard deviation of the power spectrum, the COM measures the change in frequency, the SKW is the degree of asymmetry of the spreading of a random variable around the variable mean, and the AR coefficients are based on the linear predictive model.

### 2.3. Scatter Plot

A scatter plot is normally a presentation of two variables calculated from a dataset using Cartesian coordinates, and this plot is used to visually observe the clustering performance of an algorithm and the degree of overlap among classes. The selection of two variables depends on our choice: unique variables from two channels, two separate variables from a single channel, or the first two reduced features obtained from the dimension reduction technique [29]. In our scatter plot, we used two reduced features from uncorrelated linear discriminant analysis (ULDA). Here, subject 1 data from dataset 1 was used, and each data point on the scatter plot denotes the first two reduced features from ULDA. In this case, the size of the window considered was 250 ms. Therefore, the ten different movements were presented by three hundred data points (data points × trials × movements = 5 × 6 × 10) on a single scatter plot. Moreover, the reduced feature values were normalized by using (4) to obtain a better presentation [29].

(4) $$\begin{array}{c} {\text{Normfeat}}_{i}=\frac{{\text{feat}}_{i}-\min_{i}}{\max_{i}-\min_{i}}, \end{array}$$

where max_*i*_ and min_*i*_ are the maximum and minimum values of the *i*^*th*^ feature, respectively.

### 2.4. RES Index

For evaluating the clustering performances of features, the statistical parameter RES (ratio of the Euclidean distance to the standard deviation) index is used. A higher RES index indicates higher separation among the classes and vice versa. The benefit of using the RES index is that it is independent of the classifiers used. The RES index can be evaluated as follows [68]:

(5) $$\begin{array}{c} \mathrm{RES}\text{ Index}=\frac{\bar{\mathrm{ED}}}{\bar{\sigma }}, \end{array}$$

where $\bar{E\;D}$ is the Euclidean distance between movements *p* and *q*. It is defined mathematically as

(6) $$\begin{array}{c} \bar{\mathrm{ED}}=\frac{2}{K\left(\mathrm{K-1}\right)}\sum_{\mathrm{p=1}}^{\mathrm{K-1}}\sum_{\mathrm{q=p+1}}^{K}\sqrt{{\left(m_{\mathrm{1p}}-m_{\mathrm{1q}}\right)}^{2}+{\left(m_{\mathrm{2p}}-m_{\mathrm{2q}}\right)}^{2}}, \end{array}$$

where *m* is the mean value of a feature and *k* denotes the total number of movements. The dispersion of clusters *p* and *q* is given by

(7) $$\begin{array}{c} \bar{\sigma }=\frac{1}{IK}\sum_{i=1}^{I}\sum_{k=1}^{K}s_{ik}, \end{array}$$

where *I* is the length of the feature vector.

### 2.5. EMG Pattern Recognition Method

**Figure 9:**
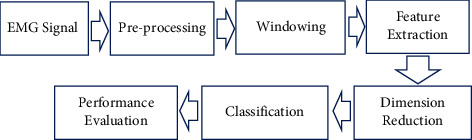
Block diagram of the myoelectric pattern recognition system.

Figure 9 shows the block diagram of the myoelectric pattern recognition system, where we employed MATLAB R2017a software (Mathworks, USA) for the EMG pattern recognition of ten-finger movements. After getting the digital EMG signal through the process in Figure 2(a) or from dataset 2, we passed the EMG signal through a digital preprocessing block using MATLAB R2017a environment where a digital bandpass filter (20 to 500 Hz) and a digital notch filter (50 Hz) were used to reduce movement artefacts, high-frequency noise [63], and power line artefacts [69]. In general, two types of windowing, i.e., overlapped and disjoint windowing, are used [70]; however, between these two, the overlapped windowing scheme offers better pattern recognition performance, but its computational cost is higher [71]. As a result, to obtain a lower computational cost with simplicity [64], we used a 250 ms disjoint windowing scheme [72] that produced 20 segments for each five-second long dataset. Hence, features were extracted using feature extraction methods (i.e., FS1, FS2, FS3, and FS4) that created a high-dimensional feature space, as mentioned in Section 2.2.2. The feature dimensionality was reduced (total classes − 1 = 10 − 1 = 9) using ULDA [73]. Now, the 9-dimensional reduced feature vectors for each feature extraction method were classified using three popular classifiers: LDA with quadratic function [74, 75], SVM with Gaussian radian basis kernel function (sigma value = 1) [76], and KNN with cityblock distance (neighbours = 3) [37]. In this performance evaluation, five trials containing 1000 samples (trials × movements × samples per trial = 5 × 10 × 20) were used as training data, and the remaining trial containing 200 samples (trials × movements × samples per trial = 1 × 10 × 20) was used as testing data. The process was repeated six times so that each of the trials was employed as testing data like 6-fold cross-validation where trial-wise performance evaluation was performed according to [38, 39]. In addition to generating a large number of training samples, the EMG pattern recognition performances during the training and testing periods were also compared, and it was found that the differences in their performances were negligible, which implied that the data were not overfitted. However, EMG pattern recognition performance was measured by accuracy, sensitivity, specificity, precision, and the *F*1 score [77, 78]. Accuracy, sensitivity, specificity, and precision describe the ability of a model to distinguish true positive and true negative movements; these metrics represent the number of positive movements correctly identified as positive, the number of negative movements correctly identified as negative, and the number of true positive movements over the positive predicted movements, respectively. Additionally, the *F*1 score combines both sensitivity and precision to find the true positive movements more precisely. These performance evaluation parameters can be defined as follows:

(8) $$\begin{array}{c} \text{Accuracy}=\frac{TP+TN}{TP+TN+FP+FN},\\ \text{Sensitivity}=\frac{TP}{TP+FN},\\ \text{Specificity}=\frac{TN}{TN+FP},\\ \text{Precision}=\frac{TP}{TP+FP},\\ F1\text{ Score}=\frac{2\times \text{Precision}\times \text{Sensitivity}}{\text{Precision}+\text{Sensitivity}}, \end{array}$$

where *TP*, *TN*, *FP*, and *FN* denote true positive movements, true negative movements, false positive movements, and false negative movements, respectively.

### 2.6. Statistical Analysis

To find the significant differences between any pairs of feature extraction methods mentioned in Section 2.2.2, a Bonferroni corrected analysis of variance (ANOVA) test was utilized with a significance level of 0.05. The obtained *p* values below 0.05 imply that the performances are significantly different. In this study, the EMG pattern recognition performances on both datasets were concatenated to construct an 18-dimensional vector (10 and 8 subjects in dataset 1 and dataset 2, respectively), and then a Bonferroni corrected ANOVA test was performed.

## 3. Results

### 3.1. Signal Observation

The EMG signals in the time domain for the ten individual and combined finger movements collected from forearm muscles are presented in Figure 4. In this figure, a time span of 250 ms was used for each finger movement. Here, no distinguishable features except amplitude were visually observed. Additionally, it was quite impossible to discriminate all movements successfully using either a single channel or a single feature. Therefore, in general, complex mathematical functions or transformations are used to enhance EMG pattern recognition performance for a minimal number of channels used.

### 3.2. Scatter Plot and RES Index

**Figure 10:**
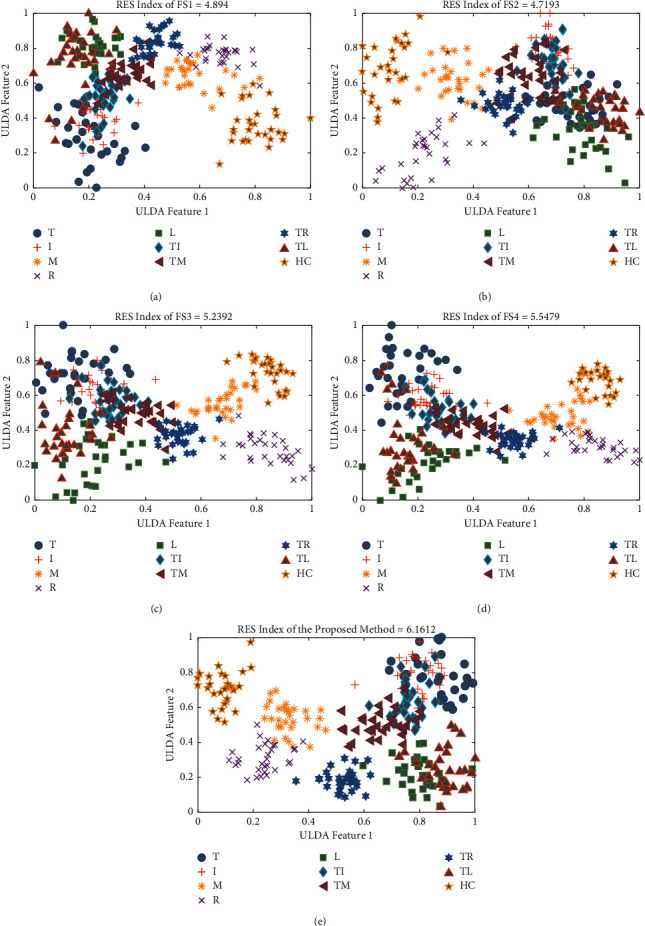
The scatter plot and RES index of different feature extraction methods for subject 1 of dataset 1: (a) FS1, (b) FS2, (c) FS3, (d) FS4, and (e) the proposed method.

The scatter plot for the different feature extraction methods mentioned in Section 2.2.2 is shown in Figure 10. In this scatter plot, all the features of the respective feature extraction methods were extracted, and the obtained high-dimensional feature space was reduced by employing ULDA. Then, ULDA features 1 and 2 were plotted in the horizontal and vertical directions, respectively. Figure 10 shows that the proposed feature extraction method provided better clustering performance than the existing feature extraction methods considered. ULDA features 1 and 2 were also used to calculate the RES index shown with the title of the corresponding scatter plot. The obtained results also indicated that the proposed feature extraction method provided the highest RES index compared to four existing feature extraction methods. Therefore, it was expected that the proposed feature extraction method could provide the best EMG pattern recognition performance.

### 3.3. EMG Pattern Recognition Performance

**Figure 11:**
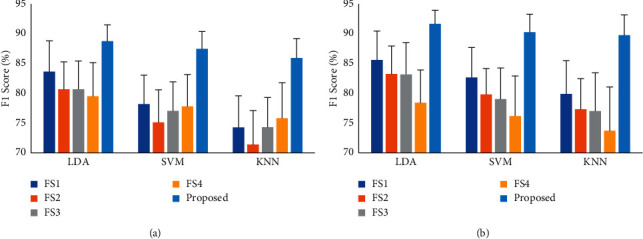
The *F*1 score of different feature extraction methods: (a) dataset 1 and (b) dataset 2.

To find the strength of the proposed feature extraction method in EMG pattern recognition performance, we compared the performances of the proposed feature extraction method with four existing feature extraction methods (FS1, FS2, FS3, and FS4). The comparison among the feature extraction methods in terms of accuracy, sensitivity, specificity, precision, and *F*1 score is shown in Table 1. The table indicates that the proposed feature extraction method achieved the highest EMG pattern recognition performance in terms of all performance evaluating parameters. In this study, the FS1 achieved the second-best performance. However, we compared this FS1 and the proposed feature extraction method and found that on dataset 1 with LDA classifier, the proposed feature extraction method improved accuracy, sensitivity, specificity, precision, and F1 score by 1.00%, 5.01%, 0.55%, 4.71%, and 5.06%, respectively; again, on dataset 2 with LDA classifier, these improvements in accuracy, sensitivity, specificity, precision, and *F*1 score were 1.18%, 5.90%, 0.66%, 5.63%, and 6.04%, respectively. The higher *F*1 score, as found, indicated that the true positive movements recognition rate was higher, which is generally expected. In addition, the *p* value between the proposed method and FS1 was less than 0.001 considering all cases (Table 2). So, the lowest *p* values indicated that the proposed method significantly improved EMG pattern recognition performance. Also, the comparison is shown graphically in Figure 11 where only the *F*1 score was used for simple presentation.

### 3.4. Performance Enhancement of Existing Feature Extraction Methods with the LMAV and NSV

**Figure 12:**
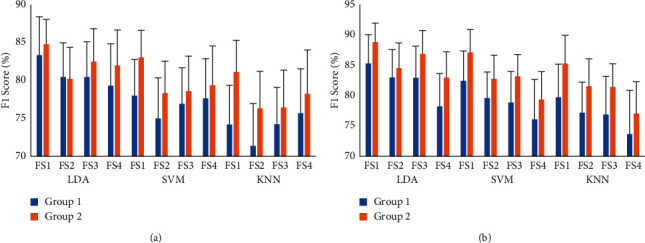
The *F*1 score enhancement of existing feature extraction methods with the LMAV and NSV: (a) dataset 1 and (b) dataset 2.

To demonstrate the effects of the proposed features, the LMAV and NSV, on EMG pattern recognition performances, the considered feature extraction methods were arranged into two groups: group 1 contained all the existing feature extraction methods (FS1, FS2, FS3, and FS4) mentioned in Section 2.2.2, and group 2 contained each of the existing feature extraction methods along with the LMAV and NSV. In this performance evaluation, we employed LDA, SVM, and KNN classifiers and their experimental results are shown in Tables 3–5, respectively. Again, to show this performance enhancement with simplicity, we considered the *F*1 score for both datasets, as shown in Figure 12. The tables implied that the LMAV and NSV enhanced the EMG pattern recognition performances of the existing four feature extraction methods with three classifiers except for a negligible degradation in the performance of FS2 with the LDA classifier on dataset 1. The performance enhancement induced by using the LMAV and NSV for each feature extraction method was also validated with a Bonferroni-corrected ANOVA. The highest *p*-value between group 1 and group 2 was 0.002 (Table 6) considering all cases except for FS2 with LDA. The obtained *p* values indicated that the LMAV and NSV significantly enhanced EMG pattern recognition performance.

### 3.5. Movement-Wise Performance Enhancement Induced by Using the LMAV and NSV

**Figure 13:**
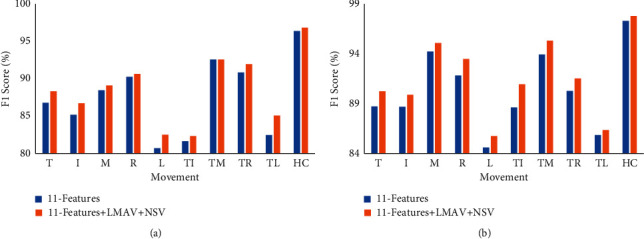
Movement-wise performance enhancement by using the LMAV and NSV using LDA classifier: (a) dataset 1 and (b) dataset 2.

To determine the movement-wise performance enhancement induced by using the LMAV and NSV, we considered the proposed feature extraction method which included the proposed LMAV and NSV and existing 11 time-domain features as described in Section 2.2.2. Furthermore, the EMG pattern recognition performance (*F*1 score) was evaluated for existing 11 time-domain features and existing 11 time-domain features along with LMAV and NSV. In this performance evaluation, we employed an LDA classifier with a 250 ms window. Figures 13(a) and 13(b) show the performance for dataset 1 and dataset 2, respectively. The figures demonstrated that the LMAV and NSV improved the *F*1 score for all the cases except for TM and HC movement of dataset 1. Moreover, all hand movements achieved a noticeably better *F*1 score (up to 2.60% and 2.30% for dataset 1 and dataset 2, respectively) for the proposed feature extraction method, the LMAV and NSV, along with existing 11 time-domain features. Also, the obtained *p* value between the overall performances of 11-features and 11-features along with the LMAV and NSV was less than 0.001 which indicated the significant improvement by the LMAV and NSV.

### 3.6. Impact of the LMAV and NSV on Performance Enhancement with a Variable Window Size

**Figure 14:**
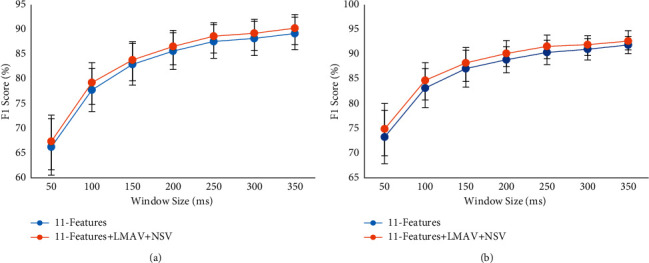
The impact of the LMAV and NSV on *F*1 score with variable window size using LDA classifier: (a) dataset 1 and (b) dataset 2.

To investigate the impact of the LMAV and NSV on the performance enhancement with variable window size, we varied the window size from 50 ms to 350 ms with an interval of 50 ms. Then, we considered the proposed feature extraction method only where the *F*1 score was evaluated for existing 11 time-domain features and existing 11 time-domain features along with LMAV and NSV using the LDA classifier, as shown in Figure 14. The figure indicated that the LMAV and NSV improved the *F*1 score for all window sizes across both datasets. In addition to the *F*1 score, the other performance evaluation parameters (accuracy, sensitivity, specificity, and precision) followed the trend of the *F*1 score. In this study, the SVM and KNN also provided a similar set of consistent results compared to those obtained under LDA. Moreover, it was also noted that the standard deviation decreased with increasing window size. A similar phenomenon was also observed for the other two classifiers. In addition, we evaluated *p*-values between the performances of 11-features and 11-features along with LMAV and NSV for each window size. The obtained *p* values between the F1 scores of 11-features and 11-features along with LMAV and NSV at various window sizes were less than 0.001 (Table 7). The smallest *p* values indicated that the LMAV and NSV significantly improved EMG pattern recognition performance for variable window sizes.

### 3.7. Impact of the LMAV and NSV on Performance Enhancement with a Variable SNR

**Figure 15:**
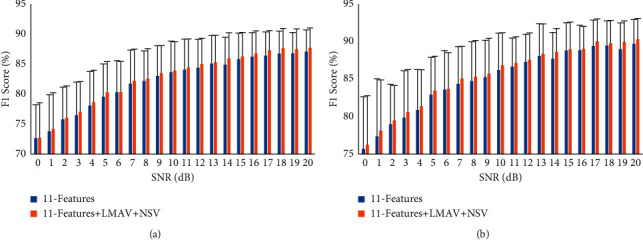
The impact of the LMAV and NSV on *F*1 with variable SNR score using LDA classifier: (a) dataset 1 and (b) dataset 2.

To find the strength of the proposed LMAV and NSV with variable SNR, we considered the proposed feature extraction method where we mixed AWGN artificially to the raw EMG signal which ranges from 0 dB to 20 dB with an interval of 1 dB [79, 80]. In this noise mixing, we employed MATLAB R2017a function (awgn) to generate an AWGN of specific dB with respect to signal and to mix the noise with the signal. Then, the EMG pattern recognition performance (F1 score) was measured for the existing 11 time-domain features and the existing 11 time-domain features along with LMAV and NSV using an LDA classifier. The EMG pattern recognition performance with standard deviation is shown in Figure 15. The following figures indicated that the EMG pattern recognition performance of time-domain features was affected by different level of AWGN; but the performance was almost stable above the SNR value of 16 dB. Another important point noted that the proposed LMAV and NSV contributed more or less to enhance the performance for all SNR values and it was valid for both datasets. Another important point noted that the proposed LMAV and NSV significantly contributed to enhancing the EMG pattern recognition performance from the SNR value of 17 dB to 20 dB since the *p* values were less than 0.01 (Table 8).

## 4. Discussion

Biosignals, such as those from electroencephalograms (EEGs), EMGs, electrocardiograms (ECGs), and photoplethysmograms (PPGs), have been widely investigated in the diagnosis of diseases and the real-time monitoring of patients [81–84]. Among these biosignals, EMG signals are widely studied as a control signal for prosthetic hands [9]. However, there is an industrial demand for providing amputees with a low-cost prosthetic hand that has reliable pattern recognition performance. Generally, the cost is minimized by reducing the number of channels and the degrees of freedom used [85]. One of the inherent reasons for compromising on the degrees of freedom in a low-cost myoelectric pattern recognition system is the least separable margin among the movements considered, especially among the movements possessing weak signal strengths [32, 48]. Therefore, the objective of this work is to obtain improved EMG pattern recognition performance with a minimal number of channels. To reach this goal, nonlinear scaling-based features, the LMAV and NSV, were proposed. The LMAV and NSV enhanced the EMG pattern recognition performance when the LMAV and NSV were grouped with each of the existing feature extraction methods considered (FS1, FS2, FS3, and FS4). This performance enhancement indicated that the LMAV and NSV added some new information to the existing feature extraction methods due to the use of nonlinear scaling on the signal amplitude rather than using the original signal amplitude used in [26]. The nonlinear scaling operation yielded higher discrimination among weak signals than strong signals and thus contributed to the performance enhancement. In this study, we also proposed a combined feature extraction method, including the proposed LMAV and NSV, along with the existing WL, WAMP, SSC, ZC, MOB, COM, SKW, and four autoregressive coefficients, which achieved the highest EMG pattern recognition performance in terms of accuracy, sensitivity, specificity, precision, and *F*1 score compared with the existing feature extraction methods [38, 39, 65, 66]. However, the proposed feature extraction method enhanced accuracy, sensitivity, specificity, precision, and *F*1 score by 1.00%, 5.01%, 0.55%, 4.71%, and 5.06%, respectively. Additionally, on dataset 2 with LDA classifier, the proposed method improved accuracy, sensitivity, specificity, precision, and *F*1 score by 1.18%, 5.90%, 0.66%, 5.63%, and 6.04%, respectively.

In this study, the LMAV and NSV were validated across two identical EMG datasets (one newly collected in our lab and one standard), where each of the datasets employed two channels and five individuals and five combined finger movements. However, these datasets employed distinct acquisition systems, electrodes, processing circuits, numbers of bits for ADC, and sampling frequencies. Again, an interesting finding was that the proposed LMAV and NSV contributed to improved EMG pattern recognition performances for both datasets; this proved the strength of the LMAV and NSV for the standard dataset and the experimental dataset that we collected from our experimental acquisition system.

Again, the LMAV and NSV yielded consistent performance enhancements over window sizes ranging from 50 ms to 350 ms, thereby ensuring the applicability of the LMAV and NSV over various window sizes. In this evaluation, we considered the maximum window size to be 350 ms since a disjoint window size higher than 250 ms did not contribute to significantly enhancing the EMG pattern recognition performance and increasing the system delay [70]. In addition, the most noticeable characteristics of the LMAV and NSV were that the proposed features mostly improved the movement-wise F1 score (up to 2.60% and 2.30% for dataset 1 and dataset 2, respectively), and this enhanced the strength of the LMAV and NSV.

It was also important to note that the proposed feature extraction method showed stable EMG pattern recognition performances across the LDA, SVM, and KNN classifiers. Therefore, the proposed feature extraction method provided an option when choosing a classifier for a given requirement.

Our study has some limitations. Dataset 1 was collected using a wet electrode; so, the noise in no movement condition was very less compared to the EMG signal during muscle contraction (average SNR lies between 5 dB and 23 dB). But, in the case of using a dry electrode, the noise may increase including power line artefacts and AWGN. So, to generate the noisy condition artificially, the EMG signal was contaminated with AWGN using MATLAB R2017a environment [79]. At low SNR values, the EMG pattern recognition performance of the proposed feature extraction method was found less in comparison with the performance at high SNR, but everywhere the proposed features (LMAV and NSV) contributed more or less to enhancing the performance. Again, the current EMG pattern recognition performance included steady-state EMG datasets (dataset 1 and dataset 2) only, but a real-time prosthetic control included both steady-state and transient condition. So, further study is required considering dry electrodes and transient condition. In addition to these, the contribution of the newly proposed LMAV and NSV in terms of pattern recognition performance will be investigated for a multichannel electrode array. A similar investigation will also be carried out for the proposed feature extraction method. In addition, the performance of the proposed feature extraction method on the other physiological will be investigated.

## 5. Conclusions

Two nonlinear scaling-based features, the LMAV and NSV, are proposed and validated across two datasets for four existing feature extraction methods and three classifiers. The experimental results indicate that the proposed features significantly enhance the EMG pattern recognition performances yielded when they are grouped with the existing feature extraction methods. It is also mentioned that the important strengths of the proposed features are stable performance enhancements on both datasets, with classifiers, under a variable window size and a variable SNR. In addition to the newly proposed features, we also propose a combined feature extraction method (the LMAV and NSV along with the existing 11 time-domain features), which achieves the best performances on both datasets and with all three classifiers. In this study, FS1 with the LMAV and NSV achieves the second-best EMG pattern recognition performance across all cases. Moreover, it is also noted that the LDA classifier provides better performance than the SVM and KNN.

## Figures and Tables

**Table 1:** The EMG pattern recognition performances of different feature extraction methods.

	Parameter	Classifier	FS1	FS2	FS3	FS4	Proposed
Dataset 1	Accuracy	LDA	96.79 ± 1.00	96.27 ± 0.88	96.21 ± 0.92	96.03 ± 1.05	97.79 ± 0.52
		SVM	95.73 ± 0.93	95.14 ± 1.06	95.53 ± 0.93	95.66 ± 1.02	97.52 ± 0.56
		KNN	94.94 ± 1.04	94.39 ± 1.12	94.95 ± 0.98	95.25 ± 1.15	97.23 ± 0.63
	Sensitivity	LDA	83.96 ± 4.98	81.36 ± 4.38	81.03 ± 4.60	80.15 ± 5.25	88.97 ± 2.58
		SVM	78.65 ± 4.67	75.70 ± 5.30	77.63 ± 4.64	78.30 ± 5.08	87.62 ± 2.80
		KNN	74.71 ± 5.19	71.94 ± 5.60	74.74 ± 4.90	76.24 ± 5.77	86.16 ± 3.13
	Specificity	LDA	98.22 ± 0.55	97.93 ± 0.49	97.89 ± 0.51	97.79 ± 0.58	98.77 ± 0.29
		SVM	97.63 ± 0.52	97.30 ± 0.59	97.51 ± 0.52	97.59 ± 0.56	98.46 ± 0.35
		KNN	97.19 ± 0.58	96.88 ± 0.62	97.19 ± 0.54	97.36 ± 0.64	98.62 ± 0.31
	Precision	LDA	85.24 ± 4.72	82.41 ± 4.28	82.55 ± 4.49	81.41 ± 4.32	89.95 ± 2.53
		SVM	79.42 ± 5.00	77.11 ± 5.13	78.83 ± 4.81	79.61 ± 5.35	88.47 ± 2.77
		KNN	75.96 ± 5.19	73.59 ± 5.40	76.27 ± 4.69	77.74 ± 5.58	87.03 ± 3.11
	*F*1 score	LDA	83.49 ± 5.13	80.56 ± 4.57	80.58 ± 4.69	79.41 ± 5.59	88.55 ± 2.72
		SVM	78.08 ± 4.84	75.06 ± 5.41	76.97 ± 4.83	77.71 ± 5.32	87.26 ± 2.88
		KNN	74.22 ± 5.26	71.38 ± 5.64	74.26 ± 4.94	75.74 ± 5.91	85.77 ± 3.24

Dataset 2	Accuracy	LDA	97.18 ± 0.94	96.73 ± 0.93	96.69 ± 1.07	95.78 ± 1.08	98.36 ± 0.45
		SVM	96.58 ± 0.97	96.00 ± 0.85	95.88 ± 1.00	95.32 ± 1.29	98.07 ± 0.59
		KNN	96.00 ± 1.08	95.54 ± 0.96	95.47 ± 1.24	94.85 ± 1.41	97.97 ± 0.66
	Sensitivity	LDA	85.88 ± 4.72	83.66 ± 4.66	83.43 ± 5.34	78.88 ± 5.40	91.78 ± 2.25
		SVM	82.88 ± 4.86	80.01 ± 4.24	79.42 ± 4.99	76.60 ± 6.44	90.34 ± 2.96
		KNN	80.06 ± 5.38	77.69 ± 4.80	77.34 ± 6.20	74.25 ± 7.04	89.83 ± 3.28
	Specificity	LDA	98.43 ± 0.52	98.18 ± 0.52	98.16 ± 0.59	97.65 ± 0.60	99.09 ± 0.25
		SVM	98.10 ± 0.54	97.78 ± 0.47	97.71 ± 0.55	97.40 ± 0.72	98.93 ± 0.33
		KNN	97.78 ± 0.60	97.52 ± 0.53	97.48 ± 0.69	97.14 ± 0.78	98.87 ± 0.36
	Precision	LDA	87.02 ± 4.69	85.26 ± 4.31	84.79 ± 4.86	80.05 ± 4.90	92.65 ± 1.99
		SVM	83.92 ± 4.92	81.51 ± 4.05	80.75 ± 5.01	78.12 ± 6.58	91.20 ± 2.68
		KNN	81.62 ± 5.41	79.11 ± 4.71	78.68 ± 6.11	75.36 ± 7.28	90.70 ± 2.95
	*F*1 score	LDA	85.55 ± 4.84	83.22 ± 4.68	83.14 ± 5.34	78.37 ± 5.50	91.59 ± 2.29
		SVM	82.63 ± 5.01	79.78 ± 4.34	79.00 ± 5.21	76.17 ± 6.70	90.19 ± 3.02
		KNN	79.87 ± 5.58	77.30 ± 5.12	77.00 ± 6.40	73.71 ± 7.33	89.70 ± 3.39

**Table 2:** The *p* values between the proposed feature extraction method and each of the existing feature extraction methods.

Parameter	Classifier	FS1	FS2	FS3	FS4
Accuracy	LDA	*p* < 0.001	*p* < 0.001	*p* < 0.001	*p* < 0.001
	SVM	*p* < 0.001	*p* < 0.001	*p* < 0.001	*p* < 0.001
	KNN	*p* < 0.001	*p* < 0.001	*p* < 0.001	*p* < 0.001

Sensitivity	LDA	*p* < 0.001	*p* < 0.001	*p* < 0.001	*p* < 0.001
	SVM	*p* < 0.001	*p* < 0.001	*p* < 0.001	*p* < 0.001
	KNN	*p* < 0.001	*p* < 0.001	*p* < 0.001	*p* < 0.001

Specificity	LDA	*p* < 0.001	*p* < 0.001	*p* < 0.001	*p* < 0.001
	SVM	*p* < 0.001	*p* < 0.001	*p* < 0.001	*p* < 0.001
	KNN	*p* < 0.001	*p* < 0.001	*p* < 0.001	*p* < 0.001

Precision	LDA	*p* < 0.001	*p* < 0.001	*p* < 0.001	*p* < 0.001
	SVM	*p* < 0.001	*p* < 0.001	*p* < 0.001	*p* < 0.001
	KNN	*p* < 0.001	*p* < 0.001	*p* < 0.001	*p* < 0.001

*F*1 score	LDA	*p* < 0.001	*p* < 0.001	*p* < 0.001	*p* < 0.001
	SVM	*p* < 0.001	*p* < 0.001	*p* < 0.001	*p* < 0.001
	KNN	*p* < 0.001	*p* < 0.001	*p* < 0.001	*p* < 0.001

**Table 3:** The EMG pattern recognition performance enhancement of existing feature extraction methods by the LMAV and NSV when LDA classifier is used.

	Parameter	Group	FS1	FS2	FS3	FS4
Dataset 1	Accuracy	Group 1	96.79 ± 1.00	96.27 ± 0.88	96.21 ± 0.92	96.03 ± 1.05
		Group 2	97.10 ± 0.62	96.23 ± 0.81	96.62 ± 0.86	96.54 ± 0.91
	Sensitivity	Group 1	83.96 ± 4.98	81.36 ± 4.38	81.03 ± 4.60	80.15 ± 5.25
		Group 2	85.48 ± 3.09	81.16 ± 4.05	83.10 ± 4.28	82.68 ± 4.55
	Specificity	Group 1	98.22 ± 0.55	97.93 ± 0.49	97.89 ± 0.51	97.79 ± 0.58
		Group 2	98.39 ± 0.34	97.91 ± 0.45	98.12 ± 0.48	98.08 ± 0.51
	Precision	Group 1	85.24 ± 4.72	82.41 ± 4.28	82.55 ± 4.49	81.41 ± 4.32
		Group 2	86.89 ± 3.09	82.66 ± 4.00	84.54 ± 4.20	84.00 ± 4.48
	*F*1 score	Group 1	83.49 ± 5.13	80.56 ± 4.57	80.58 ± 4.69	79.41 ± 5.59
		Group 2	84.94 ± 3.32	80.33 ± 4.21	82.62 ± 4.42	82.10 ± 4.75

Dataset 2	Accuracy	Group 1	97.18 ± 0.94	96.73 ± 0.93	96.69 ± 1.07	95.78 ± 1.08
		Group 2	97.87 ± 0.64	97.02 ± 0.84	97.48 ± 0.78	96.71 ± 0.86
	Sensitivity	Group 1	85.88 ± 4.72	83.66 ± 4.66	83.43 ± 5.34	78.88 ± 5.40
		Group 2	89.34 ± 3.19	85.08 ± 4.20	87.39 ± 3.91	83.53 ± 4.28
	Specificity	Group 1	98.43 ± 0.52	98.18 ± 0.52	98.16 ± 0.59	97.65 ± 0.60
		Group 2	98.82 ± 0.35	98.34 ± 0.47	98.60 ± 0.43	98.17 ± 0.48
	Precision	Group 1	87.02 ± 4.69	85.26 ± 4.31	84.79 ± 4.86	80.05 ± 4.90
		Group 2	90.49 ± 2.66	86.51 ± 3.78	88.60 ± 3.53	84.96 ± 4.07
	*F*1 score	Group 1	85.55 ± 4.84	83.22 ± 4.68	83.14 ± 5.34	78.37 ± 5.50
		Group 2	89.14 ± 3.19	84.76 ± 4.26	87.14 ± 3.95	83.19 ± 4.32

**Table 4:** The EMG pattern recognition performance enhancement of existing feature extraction methods by the LMAV and NSV when SVM classifier is used.

	Parameter	Group	FS1	FS2	FS3	FS4
Dataset 1	Accuracy	Group 1	95.73 ± 0.93	95.14 ± 1.06	95.53 ± 0.93	95.66 ± 1.02
		Group 2	96.74 ± 0.69	95.81 ± 0.84	95.84 ± 0.90	96.02 ± 1.00
	Sensitivity	Group 1	78.65 ± 4.67	75.70 ± 5.30	77.63 ± 4.64	78.30 ± 5.08
		Group 2	83.68 ± 3.43	79.06 ± 4.20	79.19 ± 4.50	80.10 ± 5.02
	Specificity	Group 1	97.63 ± 0.52	97.30 ± 0.59	97.51 ± 0.52	97.59 ± 0.56
		Group 2	97.97 ± 0.46	97.44 ± 0.54	97.44 ± 0.55	97.64 ± 0.65
	Precision	Group 1	79.42 ± 5.00	77.11 ± 5.13	78.83 ± 4.81	79.61 ± 5.35
		Group 2	84.59 ± 3.55	80.28 ± 3.91	80.21 ± 4.71	81.20 ± 5.11
	*F*1 score	Group 1	78.08 ± 4.84	75.06 ± 5.41	76.97 ± 4.83	77.71 ± 5.32
		Group 2	83.18 ± 3.62	78.43 ± 4.27	78.67 ± 4.69	79.49 ± 5.22

Dataset 2	Accuracy	Group 1	96.58 ± 0.97	96.00 ± 0.85	95.88 ± 1.00	95.32 ± 1.29
		Group 2	97.51 ± 0.76	96.63 ± 0.78	96.73 ± 0.71	95.95 ± 0.92
	Sensitivity	Group 1	82.88 ± 4.86	80.01 ± 4.24	79.42 ± 4.99	76.60 ± 6.44
		Group 2	87.53 ± 3.79	83.13 ± 3.90	83.63 ± 3.56	79.75 ± 4.59
	Specificity	Group 1	98.10 ± 0.54	97.78 ± 0.47	97.71 ± 0.55	97.40 ± 0.72
		Group 2	98.61 ± 0.42	98.13 ± 0.43	98.18 ± 0.40	97.75 ± 0.51
	Precision	Group 1	83.92 ± 4.92	81.51 ± 4.05	80.75 ± 5.01	78.12 ± 6.58
		Group 2	88.56 ± 3.49	84.38 ± 3.51	84.85 ± 3.38	81.33 ± 4.38
	*F*1 score	Group 1	82.63 ± 5.01	79.78 ± 4.34	79.00 ± 5.21	76.17 ± 6.70
		Group 2	87.40 ± 3.85	82.98 ± 3.98	83.39 ± 3.68	79.51 ± 4.67

**Table 5:** The EMG pattern recognition performance enhancement of existing feature extraction methods by the LMAV and NSV when KNN classifier is used.

	Parameter	Group	FS1	FS2	FS3	FS4
Dataset 1	Accuracy	Group 1	94.94 ± 1.04	94.39 ± 1.12	94.95 ± 0.98	95.25 ± 1.15
		Group 2	96.35 ± 0.82	95.39 ± 0.97	95.40 ± 0.99	95.76 ± 1.17
	Sensitivity	Group 1	74.71 ± 5.19	71.94 ± 5.60	74.74 ± 4.90	76.24 ± 5.77
		Group 2	81.76 ± 4.11	76.97 ± 4.84	76.98 ± 4.97	78.80 ± 5.86
	Specificity	Group 1	97.19 ± 0.58	96.88 ± 0.62	97.19 ± 0.54	97.36 ± 0.64
		Group 2	98.19 ± 0.38	97.67 ± 0.47	97.69 ± 0.50	97.79 ± 0.56
	Precision	Group 1	75.96 ± 5.19	73.59 ± 5.40	76.27 ± 4.69	77.74 ± 5.58
		Group 2	83.04 ± 3.97	78.21 ± 4.69	78.43 ± 4.71	80.10 ± 5.53
	*F*1 score	Group 1	74.22 ± 5.26	71.38 ± 5.64	74.26 ± 4.94	75.74 ± 5.91
		Group 2	81.25 ± 4.23	76.39 ± 4.93	76.52 ± 4.97	78.32 ± 5.85

Dataset 2	Accuracy	Group 1	96.00 ± 1.08	95.54 ± 0.96	95.47 ± 1.24	94.85 ± 1.41
		Group 2	97.15 ± 0.91	96.40 ± 0.88	96.36 ± 0.76	95.49 ± 1.04
	Sensitivity	Group 1	80.06 ± 5.38	77.69 ± 4.80	77.34 ± 6.20	74.25 ± 7.04
		Group 2	85.74 ± 4.54	82.00 ± 4.38	81.80 ± 3.79	77.47 ± 5.19
	Specificity	Group 1	97.78 ± 0.60	97.52 ± 0.53	97.48 ± 0.69	97.14 ± 0.78
		Group 2	98.42 ± 0.50	98.00 ± 0.49	97.98 ± 0.42	97.50 ± 0.58
	Precision	Group 1	81.62 ± 5.41	79.11 ± 4.71	78.68 ± 6.11	75.36 ± 7.28
		Group 2	86.81 ± 4.19	83.36 ± 4.24	83.17 ± 3.55	79.15 ± 4.54
	*F*1 score	Group 1	79.87 ± 5.58	77.30 ± 5.12	77.00 ± 6.40	73.71 ± 7.33
		Group 2	85.54 ± 4.76	81.73 ± 4.60	81.61 ± 3.88	77.15 ± 5.37

**Table 6:** The *p* values between group 1 and group 2.

Parameter	Classifier	FS1	FS2	FS3	FS4
Accuracy	LDA	0.001	0.210	*p* < 0.001	*p* < 0.001
	SVM	*p* < 0.001	*p* < 0.001	*p* < 0.001	*p* < 0.001
	KNN	*p* < 0.001	*p* < 0.001	*p* < 0.001	*p* < 0.001

Sensitivity	LDA	0.001	0.194	*p* < 0.001	*p* < 0.001
	SVM	*p* < 0.001	*p* < 0.001	*p* < 0.001	*p* < 0.001
	KNN	*p* < 0.001	*p* < 0.001	*p* < 0.001	*p* < 0.001

Specificity	LDA	0.002	0.199	*p* < 0.001	*p* < 0.001
	SVM	*p* < 0.001	*p* < 0.001	*p* < 0.001	*p* < 0.001
	KNN	*p* < 0.001	*p* < 0.001	*p* < 0.001	*p* < 0.001

Precision	LDA	0.001	0.069	*p* < 0.001	*p* < 0.001
	SVM	*p* < 0.001	*p* < 0.001	*p* < 0.001	*p* < 0.001
	KNN	*p* < 0.001	*p* < 0.001	*p* < 0.001	*p* < 0.001

*F*1 score	LDA	0.002	0.198	*p* < 0.001	*p* < 0.001
	SVM	*p* < 0.001	*p* < 0.001	*p* < 0.001	*p* < 0.001
	KNN	*p* < 0.001	*p* < 0.001	*p* < 0.001	*p* < 0.001

**Table 7:** The *p* values between the *F*1 scores of 11-features and 11-features along with LMAV and NSV at various window size.

Window size (ms)	*p* value
50	*p* < 0.001
100	*p* < 0.001
150	*p* < 0.001
200	*p* < 0.001
250	*p* < 0.001
300	*p* < 0.001
350	*p* < 0.001

**Table 8:** The *p* values between the *F*1 scores of 11-features and 11-features along with LMAV and NSV at various SNR.

SNR (dB)	*p* value
0	0.265
1	0.163
2	0.118
3	0.039
4	0.019
5	0.006
6	0.700
7	0.008
8	0.019
9	0.079
10	0.071
11	0.019
12	0.044
13	0.083
14	*p* < 0.001
15	0.132
16	0.044
17	0.002
18	0.010
19	*p* < 0.001
20	0.010

## Data Availability

The data used to support this study were obtained from the following website: https://www.rami-khushaba.com/electromyogram-emg-repository.html.
